# Physical and Mechanical Properties of Novel Porous Ecological Concrete Based on Magnesium Phosphate Cement

**DOI:** 10.3390/ma15217521

**Published:** 2022-10-26

**Authors:** Shuaiyu Zhao, Dongpo Zhang, Yaqiang Li, Hang Gao, Xinmiao Meng

**Affiliations:** Department of Civil Engineering, Beijing Forestry University, Beijing 100083, China

**Keywords:** magnesium phosphate cement, compressive strength, pH, void ratio, ecological concrete

## Abstract

Ecological concrete could reduce the environment impacts of the tremendous construction of infrastructures due to its favorability to plant growth. Nonetheless, the alkalinity of the ecological concrete is usually too high when using ordinary Portland cement (OPC). To solve this problem, the magnesium ammonium phosphate cement (MPC) was used to prepare a novel porous ecological concrete instead of OPC. The pH value and compressive strength of MPC were analyzed and the pore structure was evaluated. The chemical composition and morphology were investigated by an X-ray diffraction test and scanning electron microscope observation. In addition, the void ratio, compressive strength and planting-growing characteristic of MPC-based porous ecological concrete were also studied. The pH value of the MPC suspension ranged from 6.8 to 8.5, which was much lower than that of OPC. The pH value of MPC gradually increased with the increment of phosphorus/magnesium molar ratio (P/M) and the compressive strength reached a maximum value of 49.2 MPa when the P/M value was 1/4. Fly ash (FA) and ground blast furnace slag (GBFS) could improve the pore structure and compressive strength; however, the pH value was slightly increased. As the paste-to-aggregate ratio increased, the void ratio of concrete gradually decreased, while the compressive strength gradually increased. The meadow grass was planted in the MPC-based ecological concrete, and the seeds germinated in one week and showed a better growth status than those planted in the OPC-based ecological concrete.

## 1. Introduction

Concrete is the most consumed material except for water [1] and has contributed to the rapid development for human civilization. Nonetheless, the tremendous construction of buildings and infrastructures using conventional concrete have caused serious contamination to the ecological environment, leading to serious greenhouse gas emissions accompanied with a damaged ecosystem. Therefore, the development of ecological concrete especially for plant growth can not only protect the ecological environment, but also deliver significant economic and social benefits.

Porous ecological concrete is prepared by mixing low alkalinity cementitious material and single graded aggregate in a certain proportion, which has the dual attributes of safety protection and plant growth. The use of ecological concrete has a bright future in the fields of riverbank slope protection, wetland restoration and urban transformation due to its high environmental compatibility. Therefore, scholars have extensively studied on ecological concrete [2,3,4,5,6,7,8]. These studies mostly focused on the porosity and strength of ordinary Portland cement (OPC)-based porous concrete [9]. Yan et al. studied the effect of aggregate covering layer thickness on the pore structure of porous ecological concrete, and the results showed that the effective pore size was a linear correlation with the thickness of the covering layer [10]. Lian et al. studied the relationship between porosity and porous concrete strength; and the mathematical model of the relationship between compressive strength and the porosity of porous concrete was established [11]. However, the high alkalinity of OPC (pH ≈ 14) [12] was not satisfied with the normal growth environment (pH = 6–8) of the plants, and the pH values of other alternative cements, such as calcium aluminate cement [13], alkali activated cement [14], and calcium sulphoaluminate cement [15], were still too high. At present, the main methods to reduce the alkalinity are natural carbonation and alkalinity sealing [16,17]. Nonetheless, the natural carbonation method alters the microstructure of hardened cement pastes and degrades the physical and mechanical properties of concrete [18,19,20]. The sealing alkali method using epoxy resin to cover porous concrete materials also shows the disadvantages of strong toxicity and high cost. Thus, the aforementioned methods could not thoroughly solve the problem of high alkalinity of the ecological concrete.

Magnesium ammonium phosphate cement (MPC) is a new type of hydraulic cementitious material [21,22,23,24]. Comparing with OPC, MPC possesses many advantages, such as low alkalinity [25,26], higher biocompatibility [27], very rapid setting and hardening, high early strength, small drying shrinkage, hardening at low temperature, high bonding strength, high wear resistance and excellent frost resistance [28,29,30,31,32]. MPC-based porous ecological concrete can essentially solve the high alkalinity issue in OPC-based porous concrete, and can enhance the strength of porous ecological concrete due to the excellent bonding properties and high strength of MPC.

Aiming to solve the problems of high alkalinity and poor planting performance of traditional OPC-based ecological concrete, this paper attempts to use MPC to prepare porous ecological concrete, and regulates its alkalinity, strength and void ratio through mix design so as to validate its planting performance.

## 2. Materials and Methods

The magnesia oxide (MgO) powder was used to make an MPC tendon with a density of 3.46 g/cm^3^. Ammonium dihydrogen phosphate (NH_4_H_2_PO_4_), fly ash (FA), ground blast furnace slag (GBFS) and borax (Na_2_B_4_O_7_·10H_2_O) were also used in this study. The physical and chemical characteristics of MgO, FA and GBFS are shown in Table 1. The NH_4_H_2_PO_4_ was industrial-grade white crystalline powder with a purity of 98%. The borax (Na_2_B_4_O_7_·10H_2_O) in the form of white crystalline powder with a purity of 99.5% was used as a retarder. The single gradation crushed stone was used as coarse aggregate with a particle size of 19–26.5 mm.

The mixes of MPC are shown in Table 2. The ratio of NH_4_H_2_PO_4_ to MgO (P/M) here is a molar ratio. The content of borax is the mass percentage of borax to MgO. The ratio of water to the total amount of cementitious materials is represented as W/B, and cementitious materials refer to MgO, NH_4_H_2_PO_4_, FA, GBFS and borax. The content of FA or GBFS is expressed as the mass percentage of FA or GBFS to the sum of cementitious materials.

The mixes of porous ecological concrete are shown in Table 3. The paste aggregate ratio is the mass percentage of the MPC paste to coarse aggregate.

The alkalinity of porous ecological concrete is mainly determined by the alkalinity of the cementitious material. Thus, the alkalinity of MPC was tested using a pH meter to evaluate the alkalinity of the porous ecological concrete. A sample of 5 g of MPC was soaked in 50 g deionized water and was sealed to prevent carbonation. The alkalinity of soaking solution was measured by a pH meter. Each set of the samples was tested 6 times.

The compressive strength of MPC was tested according to GB/T 17671-2021 *Cement Mortar Strength Testing Method* (ISO) [33]. The compressive strength of the porous ecological concrete was tested according to GB/T 50081-2002 *Standard Test Method for Mechanical Properties of Ordinary Concrete* [34]. The size of the concrete testing sample was 100 mm × 100 mm × 100 mm and the loading rate was 0.1 MPa/s.

Void ratio is an important index of porous ecological concrete. High void ratio is beneficial to plant growth; however, it would decrease the strength. Therefore, it was very important to determine the appropriate porosity according to the specific conditions. In this paper, the porosity of porous concrete is calculated using Equation (1).

(1) $$P=1-\frac{W_{2}-W_{1}}{V}\times 100\%$$

where P is void ratio of the porous concrete, *W*_1_ is the mass of sample in air (g), *W*_2_ is the mass of sample in water (g), *V* is the volume of sample (cm^3^).

A mercury intrusion porosimetry (MIP) test was employed to measure the pore structure of MPC samples at 28 days. The hydration product of MPC samples at 28 days was measured using an X-ray diffractometer (D8 ADVANCE, Bruker, Ettlingen, Germany). The microscopic morphology of MPC samples was observed using a scanning electron microscope (SEM) (Quanta200, Fei, Tokyo, Japan).

## 3. Results and Analysis

### 3.1. MPC

The results of the alkalinity and compressive strength of MPC are shown in Table 4.

#### 3.1.1. Effect of P/M on Alkalinity and Compressive Strength

**Figure 1:**
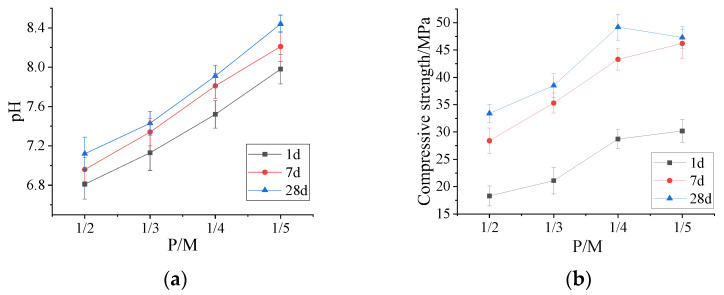
Effect of P/M on (**a**) alkalinity and (**b**) compressive strength of MPC.

Figure 1a shows that the alkalinity of MPC gradually increases as P/M decreases. When P/M is 1/2, the pH value of MPC at 1d is 6.81; however, the pH value of MPC with a P/M of 1/5 at 1d increases to 7.98. The alkalinity of MPC slightly increases over curing time; however, the pH value at 28d is still less than that of OPC (pH ≈ 14) at 28d. The pH value of MPC with P/M = 1/2 increases from 6.81 to 7.12, with the curing age increasing from 1d to 28d, respectively. It could be deduced that as the P/M ratio decreases, the increasing amount of reacted MgO results in the increase of alkalinity of MPC. With the increase of curing age, the unreacted MgO particles in the MPC matrix are further dissolved in water, resulting in a further increase in the alkalinity of MPC.

The compressive strength reaches a maximum value at a P/M of 1/4 (Figure 1b). The compressive strength of the MPC paste is mainly determined by the amount of unreacted MgO and hydration product named as struvite. When the P/M ratio is large (P/M = 1/2), the excessive cementitious products result in large amount of hydration heat, leading to thermal stress and massive cracks, which decreases its compressive strength. When the P/M ratio is too low (P/M = 1/5), the amount of struvite is very small and the unreacted MgO is abundant. Thus, the compressive strength of the MPC paste also decreases [35].

#### 3.1.2. Effect of Mineral Admixture on Alkalinity and Compressive Strength

**Figure 2:**
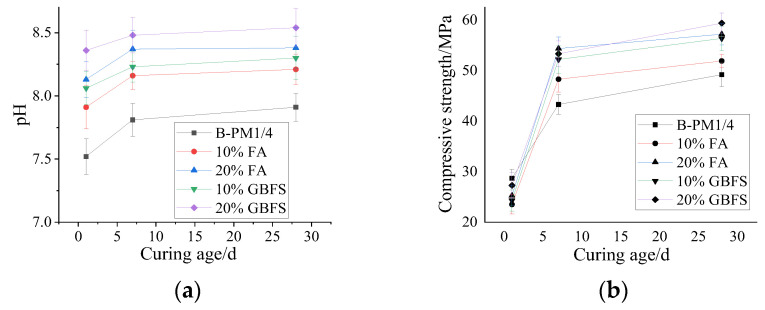
Effect of mineral admixtures on (**a**) alkalinity and (**b**) compressive strength of MPC.

The MPC paste with P/M = 1/4 was further added with FA or GBFS to investigate the effect of mineral admixture on the alkalinity and strength. The results are shown in Figure 2a,b.

The alkalinity of MPC increases with the addition of FA or GBFS (Figure 2a) and the effect of GBFS on the alkalinity of MPC is more remarkable than that of FA. The pH value of MPC without mineral admixtures at 1d is 7.52, while the pH value of MPC with the addition of 10% and 20% FA increases to 7.91 and 8.13, respectively. The pH of MPC with the addition of 10% and 20% GBFS increases to 8.06 and 8.36, respectively. The increment of the pH value mainly results from the CaO content (6.4%) and alkali content (K_2_O and Na_2_O, 1.64% in total), which react with water and increase the alkalinity of the solution. The content of CaO in GBFS is 37%, as shown in Table 1; thus, the alkalinity with the addition of GBFS is much higher than that with FA.

Figure 2b shows that the compressive strength of MPC is enhanced by the addition of FA and GBFS. The effect of GBFS on compressive strength is more remarkable than that of FA. The main reason is that FA and GBFS improve the pore structure of the MPC matrix, decrease the proportion of harmful pores in the samples, and enhance the compressive strength of MPC [36]. The GBFS particles with irregular shape show a better adhesive interface with the MPC matrix compared with the spherical FA particles. In this regard, the compressive strength of the samples with GBFS is higher than that with FA, as shown in Table 4, which is consistent with the reference [37].

#### 3.1.3. Pore Structure

**Figure 3:**
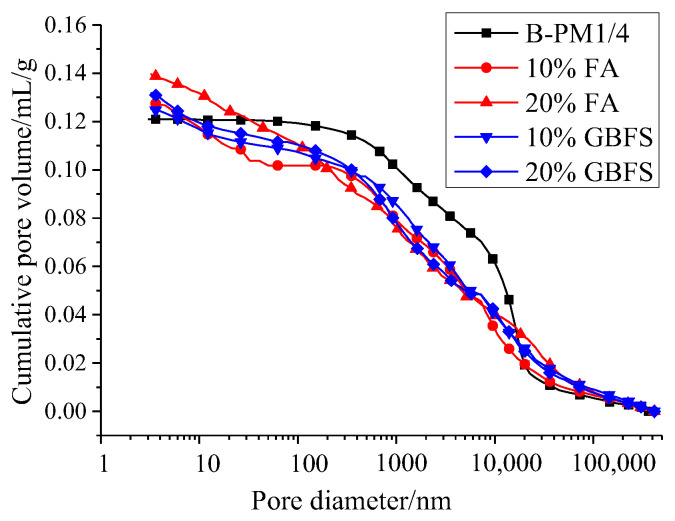
Effect of mineral admixtures on porosity of MPC.

The total pore volume of samples with the addition of FA or GBFS increases (Figure 3) mainly due to the decrease of the fluidity of the MPC paste with the addition of FA or GBFS. However, the proportion of harmful pores (>1 μm) reduced with the addition of FA and GBFS, as shown in Table 5. It can be deduced that FA and GBFS can improve the pore structure of MPC by acting as micro aggregates and reduce the proportion of harmful pores [38]. Thus, the addition of FA and GBFS can improve the compressive strength of MPC pastes.

#### 3.1.4. XRD Analysis

**Figure 4:**
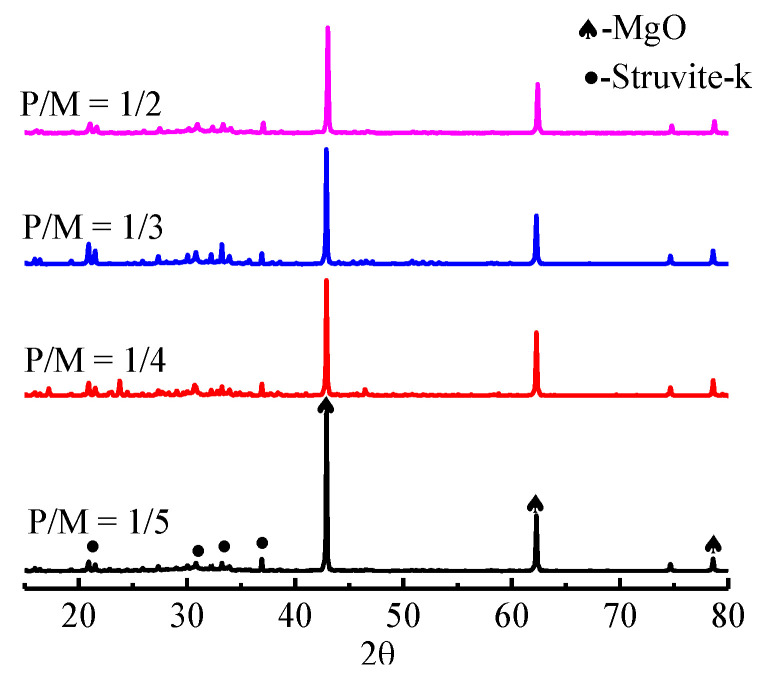
MPC components analysis of different P/M.

The X-ray diffraction (XRD) test was carried out to analyze the hydration products (Figure 4). The main components of MPC are struvite (MgNH_4_PO_4_·6H_2_O) and unreacted MgO. With the decrease in P/M, the main components are both struvite and unreacted MgO with similar XRD patterns; however, the peak intensity of MgO increases and the peak intensity of struvite gradually decreases. This is mainly because when P/M decreases, the content of unreacted MgO particles increases in MPC, affecting the compressive strength of MPC. When P/M reaches 1/5, the unreacted MgO particles content is excessive in MPC and the compressive strength of MPC begins to decrease [35].

**Figure 5:**
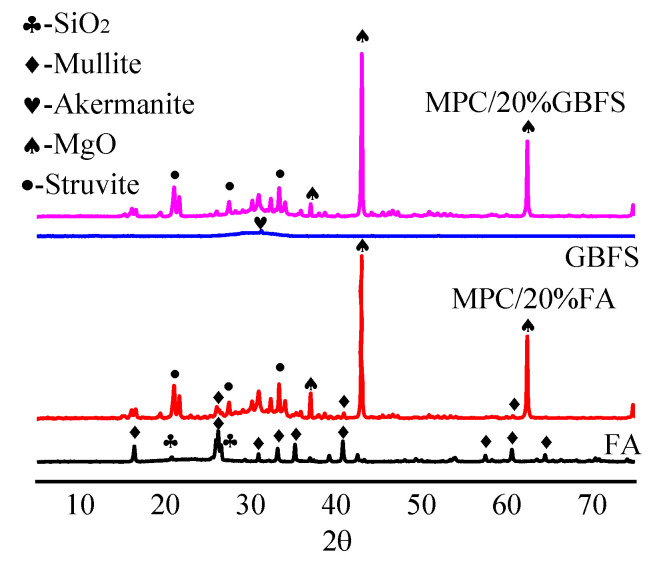
Component analysis of different mineral admixtures.

It can be seen from Figure 5 that the main crystalline phases in FA are mullite (3Al_2_O_3_·2SiO_2_) and quartz (SiO_2_), and the main crystalline phase in GBFS is akermanite (Ca_2_(Al, Mg)[(Si, Al)SiO_7_]). With the addition of FA, the main components of MPC/FA are struvite, unreacted MgO, mullite and SiO_2_ without any precipitation of new products. It is similar to the addition of GBFS; the main components of MPC/GBFS are struvite and unreacted MgO without new products. The results confirm that FA and GBFS only play a role in microporous filling without a major contribution to the chemical reaction.

#### 3.1.5. SEM Observation

**Figure 6:**
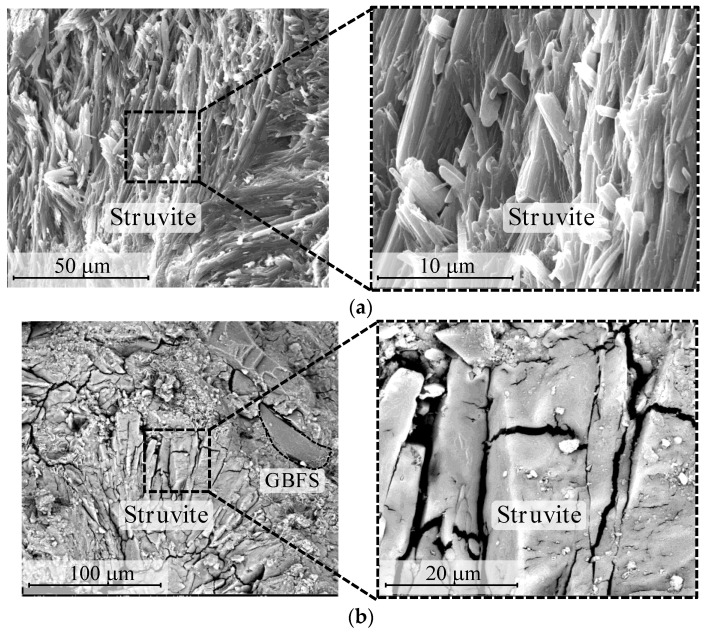

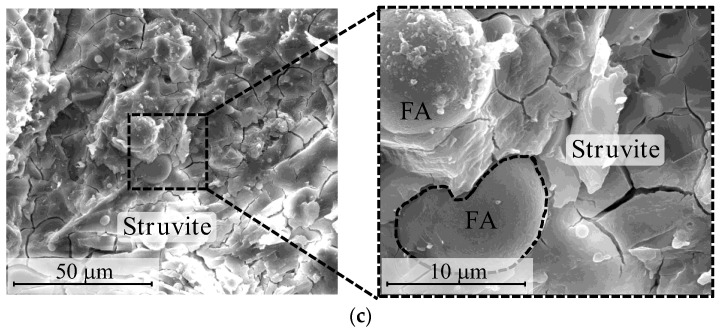
SEM observations of (**a**) MPC, (**b**) MPC/FA and (**c**) MPC/GBFS.

The microstructure of MPC was observed using SEM. According to Figure 6a, the formation of crystalline struvite and embedded unreacted MgO are identified. In Figure 6b, it can be seen that a large number of spherical FA particles are distributed in the MPC matrix. In the MPC/GBFS sample in Figure 6c, it can be seen that GBFS particles with an irregular lump are distributed in the MPC matrix. Benefiting from the irregular shape of the GBFS particles, the mechanical interlocking effect between the GBFS particles with the matrix is higher than the FA particles. Thus, the compressive strength of MPC with GBFS is higher than that with FA.

### 3.2. Porous Ecological Concrete Based on MPC

#### 3.2.1. Effect of Paste Aggregate Ratio on the Void Ratio and Compressive Strength

**Figure 7:**
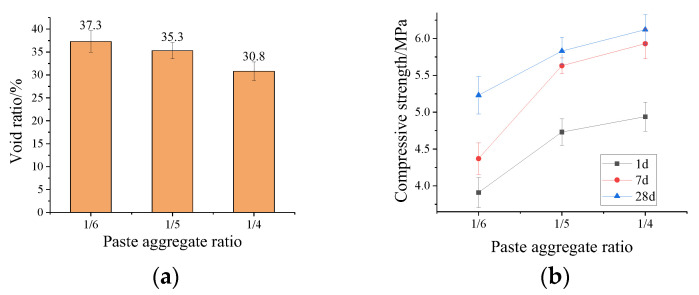
Effect of paste aggregate ratio on (**a**) void ratio and (**b**) compressive strength of porous ecological concrete.

Figure 7a shows that the void ratio of the porous ecological concrete gradually decreases as the paste aggregate ratio increases. The void ratio decreases from 37.3% to 30.8%, when the paste aggregate ratio increases from 1/6 to 1/4. It can be seen from Figure 7b that the compressive strength of the porous ecological concrete gradually increases with the increase of the paste aggregate ratio. The compressive strength of the porous concrete increases from 5.23 MPa to 6.12 MPa at 28 days, with the paste aggregate ratio increasing from 1/6 to 1/4. This leads to a thicker cover layer of aggregates and a more compact interfacial transition zone, resulting in the decrease in the void ratio and the increase in compressive strength. In addition, compared with previous research results [39], it is found that the compressive strength (28 days) of MPC-based porous ecological concrete (6.15 MPa, 5.83 MPa, 5.23 MPa) is significantly higher than that of OPC-based porous ecological concrete (1.67 MPa, 1.73 MPa, 1.14 MPa) under the same paste aggregate ratio (1/4, 1/5, 1/6).

#### 3.2.2. Effect of Mineral Admixtures on the Void Ratio and Compressive Strength

**Figure 8:**
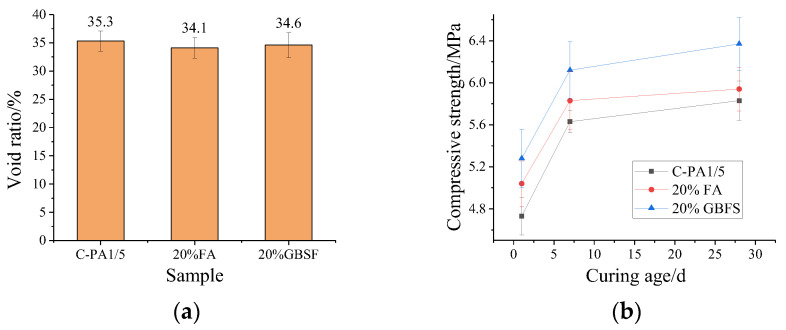
Effect of mineral admixture on (**a**) void ratio and (**b**) compressive strength of porous ecological concrete.

Figure 8a shows that both void ratios of the porous ecological concrete with the addition of 20% FA and 20% GBFS decrease slightly, which is caused by the lower density of FA and GBFS; in addition, the volume increased slightly under the same quality, leading to a slight increase in the volume of the cement paste. It can be seen from Figure 8b that with the addition of 20% FA or 20% GBFS, the compressive strength of the porous ecological concrete slightly increases. Compared to the control sample C-PA1/5, the compressive strengths with 20% FA and 20% GBFS at 28d slightly increase by 1.9% and 9.2%, respectively. The main reason is that the compressive strength of the MPC paste increases with the addition of FA and GBFS, which leads to the increment of the compressive strength of the porous ecological concrete. However, the failure of porous ecological concrete lies in coarse aggregate crushing, indicating that the strength of aggregates is a limited factor to the compressive strength. Thus, the compressive strength of porous ecological concrete is not significantly enhanced even if the strength of MPC paste is much improved.

## 4. Plant-Growing Verification of Porous Ecological Concrete

**Figure 9:**
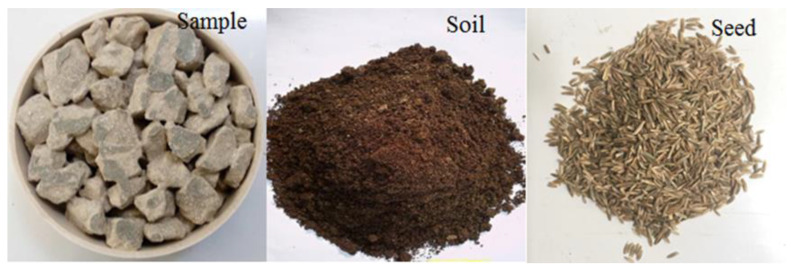
MPC-based porous ecological concrete sample, soil and seeds.

The porous ecological concrete (C-PA1/5) was used for plant-growing verification. MPC with 1/4 P/M and 20% GBFS was used for cementitious material, and OPC with 0.35 W/B was used for cementitious material for comparison. The meadow grass (*Poa pratensis* L.) was selected for ecological planting. The planting materials were composed of ordinary soil, water, a water-retaining agent and a water-reducing agent. The filling process of planting materials was based on an infiltration method. The fluidity was adjusted to the range of 180~240 mm. Then, the planting materials were poured into the pores of the porous ecological concrete. The average ambient temperature was 25 °C. The MPC-based porous ecological concrete sample, soil and seeds are shown in Figure 9.

**Figure 10:**
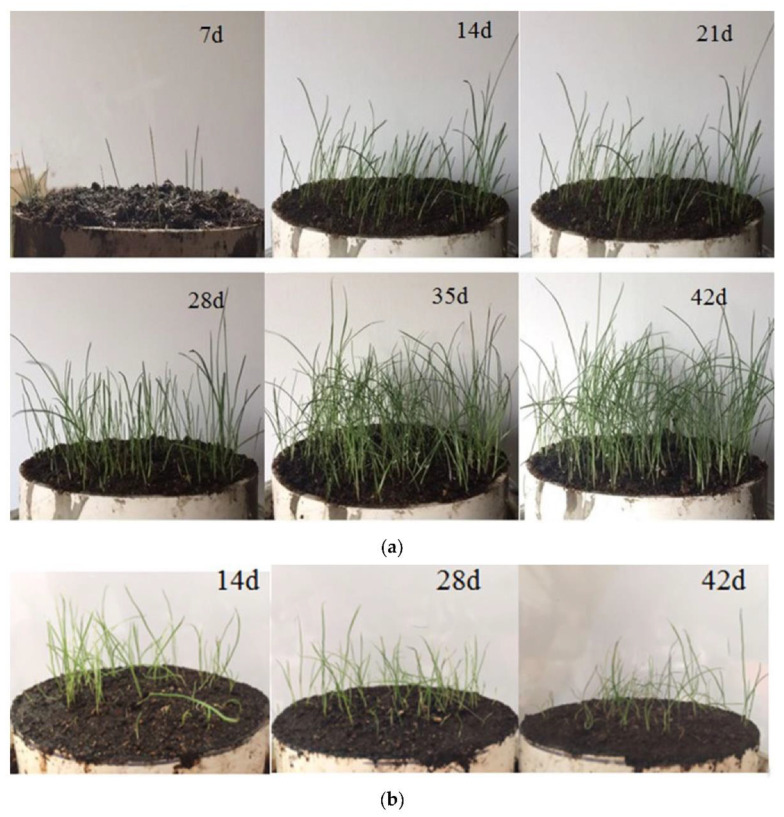
Plant growth status based on (**a**) MPC and (**b**) OPC.

The growing status is shown in Figure 10. Figure 10a shows that the seeds begin to germinate in the MPC-based porous concrete after a week, and the plants grow well and cover half of the area after 14 days. After 35 days, the plants cover almost all of the test area and the plant height reached 200 mm. The growth of the plants was flourishing with the MPC-based porous ecological concrete. Figure 10b shows that the plants have no obvious change in the OPC-based porous concrete from 14 days to 28 days, the growth of plants is poor and the covering area is only about 1/4. After 28 days, the meadow grass begins to wilt and some of them die at 42 days. The plant only reaches 100 mm in height. It is verified that the porous ecological concrete-based MPC has the potential to be applied in ecological rehabilitation projects.

## 5. Conclusions

(1) With the decrease of P/M, the alkalinity of the MPC gradually increased; however, the pH values were still much lower than the OPC. The compressive strength of MPC reached the maximum value of 49.2 MPa when P/M was 1/4.
(2) The alkalinity and compressive strength of MPC increased with the addition of FA and GBFS, and the GBFS showed more significant effects on alkalinity and compressive strength than that of FA.
(3) With the increase of the paste aggregate ratio, the void ratio of porous concrete gradually decreased from 37.3% to 30.8%, whereas the compressive strength gradually increased from 5.23 MPa to 6.12 MPa at 28 days. The compressive strength of porous concrete also increased with the addition of 20% FA and GBFS.
(4) The seeds planted in the MPC-based porous ecological concrete began to germinate after one week and grew better than those in the OPC-based ecological concrete. Thus, the MPC-based porous ecological concrete showed feasibility in ecological rehabilitation projects.
(5) At present, the setting time and cost of MPC-based ecological concrete are the main problems limiting its large-scale application. In the future, further research should be conducted in the field of research and development of environment-friendly retarders and the replacement of low-cost raw materials (such as recycled aggregate).

## Figures and Tables

**Table 1:** Physical and chemical properties of MgO, FA and GBFS.

Materials	MgO (%)	SiO_2_ (%)	Al_2_O_3_ (%)	CaO (%)	Fe_2_O_3_ (%)	TiO_2_ (%)	K_2_O (%)	SO_3_ (%)	Na_2_O (%)	Loss (%)	Specific Surface (m^2^/kg)	Density (g/cm^3^)
MgO	92	4	1.4	1.6	1.3	0	0	0	0	0	805.9	3.46
FA	0.8	48.2	33.78	6.4	5.08	1.87	1	0.75	0.6	1.45	2558	2.31
GBFS	12	32.4	12.7	37	0.4	1.34	0.4	2.27	0.5	0.7	964	2.78

**Table 2:** The mix proportions of MPC.

No.	P/M	W/B	Borax/%	FA/%	GBFS/%
B-PM1/2	1/2	0.14	5	˗	˗
B-PM1/3	1/3	0.14	5	˗	˗
B-PM1/4	1/4	0.14	5	˗	˗
B-PM1/5	1/5	0.14	5	˗	˗
B-PM1/4-FA10	1/4	0.14	5	10	˗
B-PM1/4-FA20	1/4	0.14	5	20	˗
B-PM1/4-FS10	1/4	0.14	5	˗	10
B-PM1/4-FS20	1/4	0.14	5	˗	20

**Table 3:** Physical and mechanical properties of the materials.

No.	P/M	FA%	GBFS%	Borax/%	W/B	Paste Aggregate Ratio
C-PA1/6	1/4	˗	˗	5	0.14	1/6
C-PA1/5	1/4	˗	˗	5	0.14	1/5
C-PA1/4	1/4	˗	˗	5	0.14	1/4
C-PA1/5-FA20	1/4	20	˗	5	0.14	1/5
C-PA1/5-FS20	1/4	˗	20	5	0.14	1/5

**Table 4:** pH value and compressive strength of MPC with curing ages.

Specimens	pH			Compressive Strength (MPa)		
	1d	7d	28d	1d	7d	28d
B-PM1/2	6.81 (0.15)	6.96 (0.12)	7.12 (0.17)	18.3 (1.82)	28.4 (2.32)	33.4 (1.63)
B-PM1/3	7.13 (0.18)	7.34 (0.14)	7.43 (0.12)	21.1 (2.42)	35.3 (1.82)	38.5 (2.14)
B-PM1/4	7.52 (0.14)	7.81 (0.13)	7.91 (0.11)	28.7 (1.73)	43.3 (1.94)	49.2 (2.36)
B-PM1/5	7.98 (0.15)	8.21 (0.15)	8.44 (0.09)	30.2 (2.13)	46.2 (2.64)	47.3 (1.93)
B-PM1/4-FA10	7.91 (0.17)	8.16 (0.11)	8.21 (0.12)	23.51 (1.89)	48.3 (2.57)	51.90 (1.33)
B-PM1/4-FA20	8.13 (0.14)	8.37 (0.15)	8.38 (0.16)	25.27 (1.99)	54.37 (2.27)	57.21 (2.13)
B-PM1/4-FS10	8.06 (0.13)	8.23 (0.12)	8.3 (0.17)	24.29 (2.09)	52.21 (2.71)	56.37 (2.42)
B-PM1/4-FS20	8.36 (0.16)	8.48 (0.14)	8.54 (0.15)	27.29 (2.49)	53.34 (2.52)	59.38 (2.04)

Note: the standard deviation was listed in the parentheses.

**Table 5:** Effect of mineral admixtures on pore structure of MPC.

Sample	Pore Volume/mL/g	Pore Volume Distribution/%			
		<0.01 μm	0.01–0.1 μm	0.1–1 μm	>1 μm
B-PM1/4	0.121	0.02	0.17	1.83	10.08
B-PM1/4-FA10	0.1278	1.01	1.61	2.23	7.93
B-PM1/4-FA20	0.1394	0.76	2.19	4.68	6.31
B-PM1/4-FS10	0.125	0.82	0.87	2.11	8.13
B-PM1/4-FS20	0.1317	1.22	0.91	3.27	7.77

## Data Availability

Not applicable.
